# Implementation and Evaluation of Preimplantation Genetic Testing at Vilnius University Hospital Santaros Klinikos

**DOI:** 10.15388/Amed.2022.29.2.9

**Published:** 2022-06-29

**Authors:** Eglė Stukaitė-Ruibienė, Živilė Gudlevičienė, Andrė Amšiejienė, Evelina Dagytė, Rimantas Gricius, Kristina Grigalionienė, Algirdas Utkus, Diana Ramašauskaitė

**Affiliations:** Vilnius University, Faculty of Medicine, Vilnius, Lithuania; Vilnius University, Faculty of Medicine, Vilnius, Lithuania; Centre of Obstetrics and Gynaecology, Santaros Fertility Centre, Institute of Clinical Medicine, Faculty of Medicine Vilnius University, Lithuania; Centre for Medical Genetics, Institute of Biomedical Sciences, Faculty of Medicine Vilnius University, Lithuania; Centre of Obstetrics and Gynaecology, Santaros Fertility Centre, Institute of Clinical Medicine, Faculty of Medicine Vilnius University, Lithuania; Centre for Medical Genetics, Institute of Biomedical Sciences, Faculty of Medicine Vilnius University, Lithuania; Vilnius University, Faculty of Medicine, Vilnius, Lithuania; Centre for Medical Genetics, Institute of Biomedical Sciences, Faculty of Medicine Vilnius University, Lithuania; Vilnius University, Faculty of Medicine, Vilnius, Lithuania; Centre of Obstetrics and Gynaecology, Institute of Clinical Medicine, Faculty of Medicine Vilnius University, Lithuania

**Keywords:** assisted reproductive technology, fertility, in vitro fertilization, preimplantation genetic testing

## Abstract

**Background and Objectives:**

The most effective treatment of infertility is in vitro fertilization (IVF). IVF with Preimplantation Genetic Testing (PGT) allows to identify embryos with a genetic abnormality associated with a specific medical disorder and to select the most optimal embryos for the transfer. PGT is divided into structural rearrangement testing (PGT-SR), monogenetic disorder testing (PGT-M), and aneuploidy testing (PGT-A). This study mostly analyzes PGT-SR, also describes a few cases of PGT-M. The aim of this study was to implement PGT procedure at Vilnius University Hospital Santaros Klinikos (VUHSK) Santaros Fertility Centre (SFC) and to perform retrospective analysis of PGT procedures after the implementation.

**Materials and Methods:**

A single-center retrospective analysis was carried out. The study population included infertile couples who underwent PGT at SFC, VUHSK from January 01st, 2017 to December 31st, 2020. Ion PGM platform (Life Technologies, USA) and Ion ReproSeq PGS View Kit (Life Technologies, USA) were used for the whole genome amplification. Results were assessed using descriptive statistics.

**Results:**

PGT was successfully implemented in VUHSK in 2017. During the analyzed time period, thirty-four PGT procedures were performed for 26 couples. Two procedures were performed in 2017, 7 procedures – in 2018, 13 – in 2019, and 12 – in 2020. In comparison with all IVF procedures, 2.5% procedures were IVF with PGT, a highest percentage was in 2020 (3.8% of all procedures). The main indication for PGT was balanced chromosomal rearrangements (in 85.3% cases). In all 34 cases 515 oocytes were aspirated in total, 309 oocytes were fertilized, oocytes fertilization rate exceeded 60%. A normal diploid karyotype was found in 46 (16.8%) biopsied embryos. Out of all PGT procedures, 9 (26.5%) resulted in a clinical pregnancy. Six (66.7%) pregnancies were confirmed in 2019, and 3 (33.3%) – in 2020. Three (33.3%) pregnancies resulted in spontaneous abortion, 6 (66.7%) – in delivery.

**Conclusions:**

The implementation of PGT in VUHSK was successful. The most common indication for PGT was a reciprocal translocation. Oocytes fertilization rate exceeded 60%, a normal karyotype was found less than in one-fifth of biopsied embryos. A highest clinical pregnancy rate was achieved in 2019 when almost half of women conceived, which is probably related to the experience gained by the multidisciplinary team. This is the first study analyzing IVF with PGT in Lithuania, however, the results should be interpreted with caution due to a low number of total procedures performed.

## Santrauka.

**Įvadas:**

Efektyviausias nevaisingumo gydymo būdas – pagalbinio apvaisinimo (PA) procedūra. PA su preimplantaciniu genetiniu tyrimu (PGT) leidžia identifikuoti genetiškai pakitusius embrionus ir atrinkti tinkamiausius embrionus įkelti į gimdą. Šio tyrimo tikslas – įdiegti PGT į klinikinę praktiką Vilniaus universiteto ligoninės Santaros klinikų (VULSK) Santaros vaisingumo centre (SVC) ir atlikti PGT procedūrų retrospektyvinę analizę po įdiegimo.

**Metodika:**

Atlikta retrospektyvinė analizė, į tyrimą įtrauktos VULSK nuo 2017 m. sausio 1 d. iki 2020 m. gruodžio 31 d. gydytos nevaisingos poros, kurioms taikytas PGT. Ion PGM platforma (Life Technologies, USA) ir Ion ReproSeq PGS View Kit (Life Technologies, USA) buvo naudota atlikti viso genomo sekvenavimą. Rezultatai įvertinti aprašomosios statistikos metodais.

**Rezultatai:**

PGT įdiegtas į klinikinę praktiką VULSK SFC 2017 metais. Buvo atliktos 34 PGT procedūros 26 poroms. Dvi procedūros atliktos 2017 m., 7 procedūros – 2018 m., 13 procedūrų – 2019 m., 12 – 2020 m. PGT sudarė 2,5 % visų PA procedūrų, didžiausias procentas, palyginti su visomis PA procedūromis, pasiektas 2020 metais (3,8 %). Dažniausia indikacija atlikti PGT – subalansuotas chromosomų persitvarkymas (85,3 % atvejų). Kiaušialąsčių apsivaisinimo dažnis siekė 60 %. Normalus diploidinis kariotipas rastas 16,8 % embrionų, kuriems atlikta biopsija. Iš visų PGT procedūrų 9 (26,5 %) procedūros baigėsi klinikiniu nėštumu. Šeši (66,7 %) nėštumai patvirtinti 2019 m., 3 (33,3 %) – 2020 metais. Trys (33,3 %) nėštumai baigėsi savaiminiu persileidimu, 6 (66,7 %) – gimdymu.

**Išvados:**

PGT sėkmingai įdiegtas į klinikinę praktiką VULSK. Dažniausia indikacija atlikti PGT – subalansuotas chromosomų persitvarkymas – reciprokinė translokacija. Kiaušialąsčių apsivaisinimo dažnis siekė 60 %, normalus diploidinis kariotipas nustatytas mažiau nei pektadaliui embrionų. Didžiausias klinikinių nėštumų dažnis pasiektas 2019 metais, tada beveik pusė moterų pastojo. Du trečdaliai nėštumų baigėsi gimdymu. Šiame tyrime, pirmajame Lietuvoje, apžvelgiamos PA procedūros su PGT, tačiau tyrimo rezultatai turėtų būti interpretuojami atsargiai, atsižvelgiant į mažą atliktų procedūrų skaičių.

## Introduction

Infertility is one of major health concerns nowadays and has been recognized as a public health issue by the World Health Organization (WHO) (1,2). Infertility is a disease characterized by a failure to establish a clinical pregnancy after 12 months of regular, unprotected sexual intercourse (3). It affects about 8–15% of all reproductive age couples (4,5). The most effective treatment of infertility is in vitro fertilization (IVF) which success rate is on average 25–35% and depends on the age of the couple, type and duration of infertility and other factors (6). IVF together with Preimplantation Genetic Testing (PGT) is performed to select the best embryos and to increase the pregnancy rate, to reduce the abortion rate, the multiple birth rate, the malformation rate and the rate of pointless treatments with artificial reproductive technology (ART) (7). PGT is a genetic testing procedure which allows to identify embryos with a genetic abnormality associated with a specific medical disorder known to affect one or both parents and to select the most optimal embryos for the transfer (8). PGT is divided into structural rearrangement testing (PGT-SR), monogenetic disorder testing (PGT-M), and aneuploidy testing (PGT-A) (9–11). This study mostly analyzes PGT-SR which is performed if one or both partners have chromosomal rearrangements and a high risk of passing genetic disorders to the offspring. PGT-SR decreases the risk of early pregnancy loss due to chromosome abnormalities and gives a chance to deliver a child without unbalanced structural chromosome rearrangement (12,13). In addition, this study describes a few cases of PGT-M in which both parents were carriers of pathogenic variants of autosomal recessive monogenic diseases.

The **aim of this study** was to implement PGT procedure at Vilnius University Hospital Santaros Klinikos (VUHSK) Santaros Fertility Centre (SFC) and to perform retrospective analysis of PGT procedures after the implementation. To evaluate the effectiveness of the PGT implementation, the goals of the retrospective analysis were set as: assessment of the most common indications for PGT; assessment of oocytes fertilization rate and results of genetical testing of embryos; assessment of the frequency of PGT in comparison with IVF/ICSI without PGT; and assessment of the outcomes of IVF with PGT measured by a clinical pregnancy rate.

## Materials and Methods

### The study population

A single-center retrospective analysis was carried out. The study was approved by the Vilnius Regional Committee of Biomedical Research (Approval No.2021/3-1327-804). The study population included infertile couples counseled by the multidisciplinary team and treated at SFC, VUHSK from January 01st, 2017 to December 31st, 2020. All couples that underwent PGT during this timeframe were enrolled to the study. Couples were identified at the institutional electronic database, demographic and treatment-related data were retrieved and anonymized. Data included age, type and duration of infertility or recurrent pregnancy loss, previous obstetric-gynecological history, and previous infertility treatment. The embryological data of PGT procedure for each couple included the number of oocytes retrieved, fertilization rate, the quantity and quality of embryos after intracytoplasmic sperm injection (ICSI), the number of embryos and blastomeres biopsied and transferred, indications and outcomes of PGT procedures.

### Embryos cultivation and biopsy

**Figure 1.**
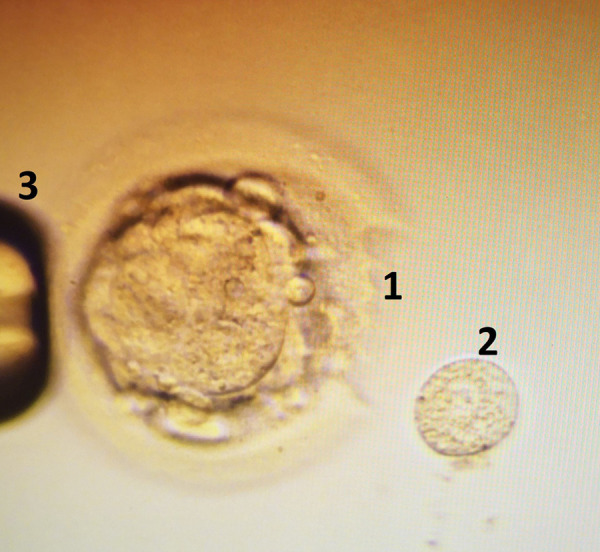
Laser assisted 4th day embryo (morula) biopsy. 1 – laser ablation of *zona pellucida*, 2 – biopsed single blastomere, 3 – holding pipette.

For PGT analyses all *embryos creation* was performed using common assisted reproduction techniques, all protocols and procedures were approved by VUHSK. Oocytes were aspirated using Cook double lumen puncture set (Cook, Australia) during the ultrasound controlled ovarian puncture for all women, directly transferred into FertiCult IVF medium (FertiPro, Belgium) and incubated until the processing in 5.5% CO2 and 37°C incubator (Astec, Japan). Two hours after aspiration, the oocytes were denuded using 135 micrometers Denuding pipette (Gynetics, Belgium) and 10% Hialuronidase (FertiPro, Belgium). Two hours after denudation ICSI procedure was performed, sperm cells were injected using RI Integra TM 3 micromanipulator (Research Instruments, UK), 35° Injection and 35° Holding micropipettes (Reproline, Germany) under an inverted Nikon Eclipse Ti microscope (Nikon, Japan). After ICSI procedure all embryos were cultivated in 50 microliters drops in the one step SAGE medium (Origio, Denmark) under the mineral oil (Irvine Scientific, USA). All embryos were revised 24, 48, and 72 hours after ICSI. According to the development speed of embryos, on day 3–4, the single embryo blastomere biopsy was performed using 1480 nm / 400 mW solid state diode laser with a pulse length range 0.005–2.0 ms / 5–2000 µs (RI Saturn 5^TM^, Research Instruments, UK) and RI Integra TM 3 micromanipulator (Research Instruments, UK) (Figure 1). Single use 50 micrometers Biopsy pipettes (Reproline, Germany) for the single blastomere biopsy were used.

All *embryo biopsies* were performed by the same embryologist. RI Viewer program (Research Instruments, UK) was used to select the best blastomere, to ablate the *zona pellucida* of the embryo and to cut the single blastomere which was quickly aspirated by the biopsy pipettes and directly transferred into the 0.2 ml microtubes with PBS/PVA (Life Technologies, USA). After the biopsy of embryos all samples were immediately transported on ice to the VUHSK Centre for Medical Genetics (CMG) for a further genetical analysis.

### Single blastomere genetical analysis

*Chromosomal rearrangement (PGT-SR) testing* of DNA from blastomere or trophectoderm biopsy was performed using the next-generation sequencing (NGS) technology according to the recommendations of the manufacture of reagents. Ion PGM platform (Life Technologies, USA) and Ion ReproSeq PGS View Kit (Life Technologies, USA) were used for the whole genome amplification, amplified DNA fragmentation and sequencing at low coverage (0.01×). Primary data analysis was performed using Torrent Suite™ Software on the Torrent server (Life Technologies, USA) and the analysis of chromosomal copy number alterations was performed using Ion Reporter™ software (Life Technologies, USA) in Thermo Fisher Cloud. This test was designed for the detection of chromosomal aneuploidy and large unbalanced rearrangements, thus ≥4.5 Mb-sized small known deletions and duplications could be specifically tested using advanced analysis workflow. Deletions and/or duplications ≥48 Mb in size were detected using a standard analysis workflow. The balanced chromosome rearrangements, uniparental disomy, some triploidies and point mutations were not detected during PGT-SR testing.

*Testing for monogenic diseases* (PGT-M for pathogenic variants in *POMK* and *SMN1* genes) was performed additionally using PGD-SEQ™ POMK Panel and Reagent Kit (Journey Genomics S.L., Spain) and PGD-SEQ™ SMN1 Panel and Reagent Kit (Journey Genomics S.L., Spain). This test allowed to combine PGT-M and PGT-SR analysis. During the first step starting from biopsied blastomeres, the whole genome was amplified using reagents provided in Ion ReproSeq PGS View Kit (Life Technologies, USA). Part of the first amplification product was used for the second region-specific amplification (PGT-M). Using appropriate PGD-SEQ™ Panel and Reagent Kit, the specific pathogenic variants and more than 130 potentially informative selected nearby polymorphisms were amplified. The PGT-M and PGT-SR libraries were sequenced using Ion ReproSeq PGS View Kit (Life Technologies, USA). The analysis of monogenic diseases was performed using PGD-SEQ software that allowed to detect carrying the disease-causing mutations embryos.

The following criteria were used for data quality evaluation: Median of the Absolute values of all Pairwise Differences (MAPD) metric value <0.3, a number of fragments (reads) mapped to the reference genome (hg19) per sample ≥50.000 (a recommended value is 100.000–300.000), the higher confidence and precision values (≥1) indicating a change in the number of copies, and reflecting correctly detected number of copies.

The results of analysis were reported as following: no DNA identified (not transferable) – no amplified DNA library after the whole genome amplification step, thus samples were not further tested; not interpreted (not transferable) – data quality did not meet quality criteria, no clear conclusion could be given; pathology (not transferable) – aneuploidy or copy number variants identified by PGT-SR testing, monogenic disease causing genotype and / or aneuploidy or copy number variants identified by PGT-M (plus PGT-SR) testing; normal (transferable) – no disease causing chromosomal aneuploidies, structural rearrangements and / or monogenic disorders were identified.

### Embryo transfer and pregnancy confirmation

After performing PGT-SR or PGT-M, on day 5 of fresh cycle, from 1 to 3 genetically normal embryos (without chromosomal abnormalities or monogenic disease) were transferred to the uterus by Cook Access Nano embryo transfer catheter (Cook, USA). If no transferable embryos were identified by PGT analysis, the possibility to transfer not tested (no DNA identified) or not interpreted but the best morphological quality embryos were discussed with the couple explaining the risk. All embryos were transferred in fresh cycle without embryo freezing. According to the Lithuanian Law for Assisted Reproduction, the maximum number of embryos which could be transferred to the uterus during one assisted reproduction cycle is three. During the time of this study more than one embryo was transferred only for women older than 30 years of age and if the morphological embryo quality was poor.

Serum human chorionic gonadotropin-β was measured 14 days after oocyte retrieval and a clinical pregnancy was confirmed by transvaginal ultrasound at 5–6 weeks. If a clinical pregnancy was achieved, a prenatal genetic testing of pregnant women was highly recommended in all cases.

### Statistical analysis

Analysis was performed and characteristics were assessed using descriptive statistics. SPSS ver. 17 (IBM Corp., Armonk, NY) was used for all quantitative analyses.

## Results

### Couples characteristics

During the period from January 01st, 2017 to December 31st, 2020, 34 PGT procedures were performed for 26 couples. In the majority (24, 70.6%) of cases, couples were undergoing PGT for the first time, in 9 (26.4%) cases – for 2nd time. One couple underwent PGT 3 times (2 out of 3 procedures were performed at SFC). In total 34 PGT procedures were performed, each procedure was analyzed as a single case.

The age of participants of the study on time the procedure was performed was on average 34 years and ranged from 28 to 42, age did not differ between males and females. The duration of subfertility varied. Time trying to conceive was 4 years on average, however, minimum duration of infertility was 2 years and maximum – 17 years. Ten (38.5%) couples (14 (41.2%) cases) already had biological children, one of them was diagnosed with type 1 spinal muscular atrophy, another one – with 21 chromosome trisomy. One male had a daughter diagnosed with a chromosomal abnormality from a previous marriage. The BMI of females was 24.6 kg/m^2^ on average. Almost one third of women (32.4%) was overweight, 4 (11.4%) were obese.

### Genetic counseling of the couples

Before IVF treatment all couples were counseled by the multidisciplinary team regarding genetical testing. A variety of chromosomal abnormalities identified to PGT patients is listed in Table 1. The most common indication for PGT was structural chromosomal rearrangements in 29 (85.3%) cases. Structural rearrangements included 6 (20.7%) Robertsonian translocations, 22 (75.9%) reciprocal translocations, and one (3.4%) chromosome inversion. Other indications were sex chromosome abnormality (2 cases, 5.9%), monogenic disease carriers (2 cases, 5.9%), and a high spontaneous chromosomal mutation risk in 1 (2.9%) case. As for monogenic disease carriers, in one case female and male were heterozygous carriers of *POMK* gene pathogenic variant c.136C>T, p.(Arg46Ter), in second case – female and male were heterozygous carriers of *SMN1* gene 7-8 exons deletion. In one case identified as a high spontaneous chromosomal mutation risk, a female patient already had a child with trisomy 21, she also had two miscarriages and a termination of pregnancy due to trisomy 18.

### Oocytes fertilization, embryos development and genetical testing

In all 34 cases 515 oocytes were aspirated in total, on average 15 (from 1 to 33) oocytes per case. After ICSI was performed, 309 oocytes were fertilized, on average 9 (from 1 to 20) per case. Fertilization rate exceeded 60%. Good quality embryos (274, 88.7% of all fertilized) were biopsied and sent for DNA amplification.

**Table 1.** Variety of chromosomal abnormalities identified to PGT patients.

Structural chromosomal rearrangements		PGT, n
Robertsonian translocation		6
1	45,XY,der(13;14)(q10;q10)	4
2	45,XX,der(13;14)(q10;q10)	1
3	45,XY,der(14;21)(q10;q10)	1
Reciprocal translocation		22
1	46,XY,t(9;12)(q32;q22)	2
2	46,XY,t(8;13)(p23.3;q14.1)	2
3	46,XY, t(3;8)(q25;p23)	1
4	46,XX,t(15;19)(q24;q13.3)	2
5	46,XX,t(13;18)(q12.3;q21.3)	2
6	46,XY,t(1;15)(p36.2;q15)	2
7	46,XX,t(4;8)(q13;q11.23)	1
8	46,XX,t(X;3)(p22.1;q21)	2
9	46,XY,t(10;15)(q24;q26.1)	2
10	46,XX,t(5;6)(p12;q14)	1
11	46,XX,t(6;14)(q14;q32.2)	1
12	46,XX,t(8;9)(q22.1;q13)	1
13	46,XY,t(7;13)(p13;q22)	1
14	46,XX,t(2;6)(p23;q21)	1
15	46,XX,t(13;14)(q14.2;q11.2)	1
Inversion		1
46,XY,inv(1)(q21q42)		1
**Sex chromosome abnormality**		2
45,X[4]/46,XY[30]		1
45,X[27]/47,XYY[16]/46,XY[7]		1

In more than half of cases (18, 52.9%) biopsies were performed on day 3 embryos, in 16 (47.1%) cases – on day 4 embryos. Out of all biopsied embryos, further developed 190 (69.3%), on average 6. Genetic analysis showed that normal diploid karyotype was found only in 46 (16.8%) biopsied embryos, 112 (40.9%) embryos had chromosomal aneuploidies, 65 (23.7%) embryos were not interpreted due to chaotic genomic imbalances, for 51 (18.6%) embryos no DNA was identified after the whole genome amplification step. In more than third (13, 38.2%) PGT procedures none of embryos had normal diploid karyotype. In 25 (73.5%) cases blastocysts on 5th day of development were transferred to uterus. In 4 (11.8%) cases no embryos were developing after biopsy, therefore, the transfer was not performed. During the majority of procedures (15, 44.1%) one embryo was transferred, in 8 (23.5%) cases – two embryos, and in 2 (5.9%) cases – 3 embryos. In four cases genetically uninterpreted but the best morphological quality embryos were transferred, the risk was explained to the couples and the signed permission for this type of transfer was received. One female conceived after transferring an uninterpreted embryo, a healthy girl (karyotype 46,XX) was born.

### The outcomes of PGT

**Figure 2.**
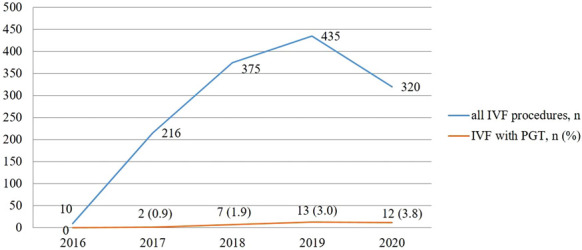
A comparison of total number of IVF procedures with IVF with PGT.

The first PGT at SFC was performed in September 2017. To compare with all IVF procedures performed at SFC, only 2 procedures were performed in 2017 (0.9% out of 216 procedures), 7 – in 2018 (1.9% of 375 procedures), 13 – in 2019 (3.0% of 435 procedures), and 12 – in 2020 (3.8% of 320 procedures) (Figure 2).

In 2017 and 2018 none of the procedures resulted in a clinical pregnancy. Almost half (6, 46.2%) procedures performed in 2019 resulted in a clinical pregnancy, and in 2020 – 3 (25%). Out of all PGT procedures, 9 (26.5%) times embryos were not transferred to uterus, 15 (44.1%) procedures were unsuccessful, 1 (2.9%) time biochemical pregnancy was diagnosed, 9 (26.5%) procedures resulted in a clinical pregnancy. Six (66.7%) clinical pregnancies were confirmed in 2019, 3 (33.3%) – in 2020. Out of 9 clinical pregnancies, 3 (33.3%) pregnancies resulted in a spontaneous abortion, 6 (66.7%) pregnancies – in delivery. Four newborns were delivered in 2019 (in one case twins), and 3 newborns – in 2020.

## Discussion

PGT was successfully implemented in VUHSK in 2017. During the time period from 2017 to 2020, 34 PGT procedures were performed. During the same time period 1346 IVF/ICSI procedures were performed in total: 216 in 2017, 375 in 2018, 435 in 2019, and 320 in 2020. Thirty-four PGT cases make only 2.5% of all IVF procedures.

The most common indication for PGT-SR in our study was a structured chromosomal rearrangement – reciprocal translocation, in 22 (64.7%) of cases. It is a typical finding as reciprocal translocations together with Robertsonian translocations and inversions are the most common chromosomal structural abnormalities (13). The genetic testing in this study was performed by NGS, it allowed to identify and screen for embryos with reduced viability such as mosaic embryos and those with partial aneuploidies or triploidy. Other studies revealed that NGS improves pregnancy outcomes versus array comparative genomic hybridization (14), there was a tendency towards a higher live birth rate for NGS testing in comparison with fluorescence in-situ hybridization and microarray comparative genomic hybridization (15).

As it was mentioned, all embryos were transferred in fresh cycles in our study. Although we could not find data comparing outcomes between fresh and frozen cycles when PGT-SR or PGT-M was applied, it is known that time to a clinical pregnancy is likely to be shorter using fresh embryo transfer during conventional IVF/ICSI than in a ‘freeze all’ strategy (16). More than one or double embryo transfer (DET) demonstrates a superior pregnancy and live birth rate, however, it is associated with a significantly higher risk of multiple gestations and increased risk for maternal and neonatal morbidity (17,18). As it was noticed, a maximum number of embryos allowed by Law in Lithuania to transfer during one cycle is three. In order to achieve the best pregnancy rate after IVF/ICSI, more than 1 embryo is usually transferred in Lithuanian clinics for assisted reproduction. In our study more than one embryo was transferred in more than half of cases, however, these cases included women older than 30 years of age and poor morphological quality embryos. Three embryos were transferred in two cases only – in one case a procedure was unsuccessful, in another case it resulted in a twin delivery.

In our study the outcomes of PGT were evaluated by a clinical pregnancy rate only. However, due to a small number of cases included in the analysis, the another important quality criteria – live birth rate – was not evaluated (15,19,20). Other evaluation method of PGT outcomes is ongoing pregnancy rate at 20 weeks’ gestation per embryo transfer (21). According to the retrospective analysis performed we could state that the best outcomes of PGT measured by a clinical pregnancy rate were achieved in 2019 when almost half (46.2%) of procedures resulted in clinical pregnancies. Regardless of the low number of cases the increased clinical pregnancy rate is probably related to the team of embryologist and medical geneticists gaining more experience – medical geneticists from VUHSK CMG successfully participated in the GenQA external quality assessment for PGT for chromosomal rearrangements. According to other studies, the clinical pregnancy rate after PGT varies from 25% to 59% and depends on many factors such as a type of chromosomal rearrangement, a type and method of PGT used, age and other characteristics of partners and the experience of the team (13,15,19,25,26). Outcomes of the procedures performed at SFC in 2019 was similar to the results obtained at other centers. However, in 2020 the quality rate dropped down. It is important to notice that from March to May of 2020 SFC was closed due to the lockdown as a consequence of COVID-19 pandemic. It is likely this contributed to a decrease in a total number of IVF/ICSI procedures thus an increase in a percentage of IVF/ICSI with PGT in comparison with total IVF procedures (Figure 2).

The Law for the Assisted Reproduction was enforced by the Lithuanian Parliament on 01/01/2017, therefore, studies investigating assisted reproductive technology (ART) in Lithuania were very limited. The first public University Hospital ART center in Lithuania, SFC at VUHSK was established in 2016 and the first PGT at SFC was performed in September 2017. The first successful live birth after PGT in Lithuania was achieved in 2019 (27). According to the Law, PGT could have been applied only after a multidisciplinary genetic counselling for couples with a high risk for passing genetic disorders to their offspring. Routine genetic testing of all *in vitro* created embryos is prohibited by the Law, explaining a low percentage of PGT procedures compared with all IVF procedures. Some other centers perform PGT for all IVF/ICSI patients as a routine genetic testing. Although limited evidence suggests that PGT-A could be beneficial in the ≥38 years old population (22), and PGT-A use is associated with improved live birth rates in couples with recurrent pregnancy loss undergoing frozen embryo transfer (FET) (23), a value of PGT as an universal genetic screening for all IVF patients has yet to be determined and remains controversial (20,24). However, routine genetic testing would have contributed to a higher number of PGT procedures in our study and probably to a higher percentage of clinical pregnancies and live births, as well as more experience gained by the multidisciplinary team.

On account of a low total number of procedures only a descriptive data analysis was carried out since the results of statistical tests could be misleading in our study. A low number of procedures and live births could be recognized as a major limitation of the study, however, PGT procedure was successfully implemented, and this is the first time when data regarding PGT in Lithuania was analyzed, therefore, a ground for a further research was prepared. The comparison group in future studies could include patients who underwent IVF/ICSI without PGT and outcomes of procedures could be compared as it was done in other studies (19,22). Results of this study will provide an insight to a further clinical practice at SFC and will contribute to a better outcomes of ART procedures in Lithuania.

## Conclusions

To summarize, PGT was successfully implemented in VUHSK after the adoption of Lithuanian Law for the Assisted Reproduction. During the evaluation period the most common indication for PGT-SR was a balanced chromosomal rearrangement – reciprocal translocation. Oocytes fertilization rate exceeded 60%, however, a normal diploid karyotype was found less than in one-fifth of biopsied embryos. Out of all IVF/ICSI procedures, PGT contained only 2.5% which is related to the prohibition of routine genetic testing of embryos by Law in Lithuania. Out of all PGT procedures more than a quarter resulted in a clinical pregnancy. The clinical pregnancy rate was highest in 2019 when almost half of women conceived. Increased clinical pregnancy rate could be related to the experience gained by the multidisciplinary team. This is the first study analyzing and systematizing PGT procedures in Lithuania. Nevertheless, the results should be interpreted with caution due to a low number of total procedures performed.
